# Caryocar brasiliense oil improves cardiac function by increasing Serca2a/PLB ratio despite no significant changes in cardiovascular risk factors in rats

**DOI:** 10.1186/s12944-017-0422-9

**Published:** 2017-02-08

**Authors:** Lidiane Guedes Oliveira, Lauane Gomes Moreno, Dirceu Sousa Melo, Liliane Vanessa Costa-Pereira, Mayara Medeiros de Freitas Carvalho, Paulo Henrique Evangelista Silva, Ana Maria Alves, Flávio de Castro Magalhães, Marco Fabrício Dias-Peixoto, Elizabethe Adriana Esteves

**Affiliations:** 1 Programa Multicêntrico de Pós-Graduação em Ciências Fisiológicas, Sociedade Brasileira de Fisiologia (SBFis) – Universidade Federal dos Vales do Jequitinhonha e Mucuri – UFVJM, Rodovia MGT 367 – Km 583, n° 5000, Alto da Jacuba, Diamantina, MG Brazil CEP: 39100-000; 2 Programa de Pós-Graduação em Ciências Biológicas, Universidade Federal de Ouro Preto - Campus Universitário, Morro do Cruzeiro, Ouro Preto, MG Brazil CEP: 35400-000

**Keywords:** *Caryocar brasiliense*, Monounsaturated fatty acids, Oleic acid, Carotenoids, Cardiac function, Cardiovascular disease

## Abstract

**Background:**

*Caryocar brasiliense* (*pequi*) oil is high in monounsaturated fat acids (MUFA), especially oleic, and in carotenoids, which have been associated with protection against cardiovascular disease. However, this food is poorly studied in this context, especially in the cardiac function. Therefore, we investigated the effects of a long-term intake of *pequi* oil in systemic cardiovascular risk factors and in the ex vivo cardiac function of rats.

**Methods:**

Previously, we determined fatty acids and carotenoids in *pequi* oil. Next, male rats were divided in C – control group feed a standard diet, and PO – *pequi* oil group fed the same diet added *pequi* oil (+2.25 g.100 g^−1^). After 15 weeks, plasma lipids, glucose, insulin, blood pressure, heart rate, hepatic lipids were accessed and visceral fat pads were harvested. Hearts were used for the ex vivo cardiac function, histologic assays, SERCA2a and phospholanban (PLB) determinations.

**Results:**

In agreement with scientific data, *pequi* oil had expressive amounts MUFA, especially oleic acid, and carotenoids. Hepatic triglycerides (TG) were reduced by *pequi* oil intake (*p* < 0.05). All others cardiovascular risk factors were not changed. The intrinsic heart rate was lower in PO group (*p* < 0.05). SERCA2a content was higher in this group (*p* < 0.05), without affecting PLB. Also, SERCA2a/PLB ratio increased in PO group (*p* < 0.05).

**Conclusion:**

*Pequi* oil intake improved cardiac function ex vivo, despite no significant changes in systemic cardiovascular risk factors. The higher lipid offer in *pequi* oil diet, its composition in oleic acid and carotenoids could be related to those effects.

## Background

Cardiovascular diseases (CVD) are responsible for 30% of all deaths worldwide each year [1] being the high intake of lipids one of the major modifiable risk factor in the etiology of these diseases [2]. Therefore, it has been indicated the amount and quality of dietary lipid as a main guideline target for reducing mortality and morbidity by those diseases [2, 3]. In this context, scientific interest has been particularly directed to the main fatty acids found in foods: trans, saturated (SFA), polyunsaturated (PUFA) and specially of our interest, monounsaturated (MUFA) fatty acids [4].

Indeed, in foods, some vegetable oils have instigated interest because their content of MUFA, and, or, PUFA, which have been associated to health benefits [5]. In this perspective, *pequi* oil has a potential for reducing cardiovascular risk since it is high in MUFA, beyond others bioactive compounds that also have been associated to cardiovascular protection.

This oil is extracted from the *Caryocar brasiliense* fruit *(pequi*) and its major fatty acid is oleic (54%), the main MUFA in the diet [6]. Scientific evidences suggest that MUFA are associated to coronary heart disease prevention [7], by favorably improving blood lipids [8], reducing blood pressure [9], and modulating insulin sensitivity and glycemic control [10]. Oleic acid is pointed out as the main responsible for those effects. In addition, MUFA has also been associated with improvements in cardiac function, since they are main components of cardiomyocyte phospholipid membranes, ameliorated endothelial function and reduced both apoptosis of vessel smooth cells and cardiomyocytes [11, 12].

This oil also has high content of palmitic acid (35%), a common SFA in animal foods [13]. SFA are more easily oxidized by cardiomyocytes and they were associated to improvements in systolic cardiac function [14]. Moreover, fatty acids in general, are cardiac important sources of energy, since almost 50-70% of ATP used by those cells, is derived from acetyl coenzyme A (Acetil CoA), the product of the fatty acids β-oxidation [15].

*Pequi* oil also has a substantial amount of carotenoids (8,10 mg.100^−1^g), especially violaxanthin, lutein, zeaxanthin, β-cryptoxanthin, neoxanthin and β-carotene [16, 17]. These compounds also have been related to cardiovascular risk reduction. They are potent antioxidants in biological systems and protect against oxidative damage [18]. Also, Csepanyi et al. [19] demonstrated that, in lower doses, these bioactive compounds improved cardiac function in rat isolated hearts, by langendorff system.

Therefore, *pequi* oil is a potential cardiprotector food. It could favorably modulate cardiac function, and improve systemic cardiovascular risk factors. However, although there are many evidences from its chemical components related to cardiovascular health, this food has been poorly studied in this context. To our knowledge, there are some research showing healing [20], chemopreventive [21, 22], anti-mutagenic [23], antioxidant [24], anti-inflammatory, antihypertensive [25] and anti-cancer [26, 27] properties of *pequi* oil in humans or animal models. In addition, most of the information has not been obtained from its intake. In addition, so far, there is no research regarding the effects of this oil directly in cardiac function.

Therefore, the aim of this study was to evaluate the effects of a long term *pequi* oil intake in systemic cardiovascular risk factors and in the ex vivo cardiac function of rats. We also explored the involvement of key proteins that modulate cardiac contractility and relaxation.

## Methods

### Preliminary analysis of *pequi* oil

*Pequi* oil was purchased from the local market in Diamantina-MG, Brazil. Previously to the rat study, fatty acids were determined by gas chromatography (CGC Agilent 6850 Series GC System) according to the AOCS Ce 1–62 method [28]. Total amount of carotenoids was determined according the AOAC Official Methods of Analysis [29], using a spectrophotometer (Specord 210, model Analytikjena), at 450 nm.

### Bioassay design

Sixteen male *Wistar* rats, 25 days old, were housed in individual stainless steel cages and kept in a room at 22 ± 2 °C and at a 12 h light/dark cycle, with free access to food and water during all experimental period (15 weeks). In the first day, all animals were randomly assigned into two treatments: C – control, fed commercial chow (*n* = 8) (RhosterLab®, energy density: 328.06 Kcal.100 g^−1^) or PO – *pequi* oil, fed commercial chow added *pequi* oil (*n* = 8). *Pequi* oil was added to increase by 50% the lipid chow content(+2.25 g.100 g^−1^), which also increased its energy density up to 348.31 Kcal.100 g^−1^.

During the experiment, body weight and food intake were monitored for Feed Efficiency (FER(g/g) = body gain/food intake) and Energy Efficiency (EER (g/Kcal) = body gain/energy intake) ratios [30]. In the last day, overnight fasted animals were anesthetized (quetamin + xilazin/50 mg/kg + 10 mg/kg), and their nose-anus length were measured for Lee Index (LI) calculation (LI = [^3^√body wheight (g) ÷ nose = anus lenght(cm)] ×10) [30].

After that, all animals were euthanized by decapitation for blood, livers, hearts, and tissue harvesting. All retroperitoneal and epididimal fat pads were removed and weighted in an analytical scale (Shimadzu AX 200) for the Adiposity Index calculation (AdI% = (epididimal pad + retroperitoneal pad)/body weight – (∑epididimal pad + retroperitoneal pad) *100) [31]. Blood was centrifuged in heparinized tubes to obtain plasma, which were aliquoted in eppendorf tubes and kept at -80^o^ C until analysis.

### Cardiovascular risk factors assays

Fasted plasma glucose levels (GLU) were measured by a commercial kit, according the procedures recommended by the manufacturer and using a semi-automatic biochemical analyzer (PIOWAY-3000). Fasted plasma insulin (INS) was determined using a commercially available Enzyme-Linked Immunosorbent Assay kit – ELISA (Linco Research Inc., St. Louis, MO, USA) and a micro-plate reader (Spectra MAX 190, Molecular Devices, USA). Insulin resistance was accessed by the homeostasis model assessment of insulin resistance (HOMA-IR index), from fasted glucose and insulin levels according to Matthews et al. [32].

Total plasma cholesterol (CHOL), high-density lipoprotein cholesterol (HDL-C), Low-density lipoprotein cholesterol (LDL-C) and triglycerides (TG) levels were determined using commercial kits according to the specifications of the manufacturer and a semi-automatic biochemical analyzer (PIOWAY-3000). Liver samples were oven-dried (60 °C ± 2 °C for 72 h), and their lipids were extracted according to Folch et al. [33]. CHOL and TG levels were determined using commercial kits, according to specifications of the manufacturer, and using a semi-automatic biochemical analyzer (PIOWAY-3000).

Systolic blood pressure (BP), as well as heart rate (HR), were measured at the last week prior to the end of the experiment by the tail-cuff plethysmography method (MLT1020PPG IR Plethysmograph, PowerLab). Additionally, the double product index was calculated using systolic blood pressure and heart rate values, as an indicative of cardiac work [34].

### Ex vivo analysis and Langendorff preparation

In the last day of the experiment, animals were anesthetized (quetamin + xilazin/50 mg/kg + 10 mg/kg) and decapitated 10–15 min after a 400 IU intraperitoneal heparin injection. Hearts were perfused in a Langendorff apparatus (ML785B2, ADInstruments) and left ventricular pressure (± dP/dt) was continuously recorded according to the Langendorff technique [35], using the Labchart 8 software. Systolic tension, diastolic tension, coronary flow, heart rate, and ± dT/dt values were the average of the recorded 30 min. All the ± dP/dt measurements were normalized to heart weight.

### Heart/body weight ratio and histologic analysis

At the end of the cardiac function analysis, wet heart weights were recorded, normalized for the body weight, and expressed as muscle mass index (mg.g^−1^), according to Almeida et al. [36].

For cardiomyocyte diameter, hearts were fixed in 4% Bouin fixative solution, embedded in paraffin, and sectioned to a5 um thickness. To determine myocyte cross-sectional area, heart sections were stained with hematoxylin and eosin and examined at 40× magnification. Only myocytes longitudinally cut with the nucleus centrally located in the cell and with cellular limits visible were used. The cross-sectional diameter (um) of the myocytes was traced using ImageJ software (National Institutes of Health), and determined by averaging 50 to 100 individual cardiomyocytes within the ventricular free wall over 5 or 6 sections per animal. A single investigator blinded to the experimental groups performed the analysis.

### Western blotting

Total protein content of left cardiac ventricles was quantified by means the Bradford protein assay [37]. Protein (80 μg) was loaded onto a 10% polyacrylamide gel for electrophoresis. After electrophoresis, proteins were transferred to a PVDF membrane, blocked with a phosphate-buffered saline, containing 0.1% Tween 20 and 5% bovine serum albumin. Membranes were incubated overnight at 4 °C with the following primary antibodies: monoclonal sarcoplasmic reticulum Ca2 + −ATPase isoform 2 (SERCA2a) (1:1000 dilution; Cell Signaling); monoclonal glyceraldehyde 3-phosphate dehydrogenase (GAPDH) (1:3000 dilution; Cell Signaling); and phopholamban (PLB) (1:6000 dilution; Cell Signaling). Thereafter, a monoclonal anti-rabbit or anti-mouse secondary antibody conjugated with peroxidase (1:4000 dilution, Cell Signaling) were used. Immunodetection was carried out using enhanced chemiluminescence (AmershamBiosciences), and protein levels were expressed as a ratio of optical densities.

### Statistics

The statistical analyses were carried out using the Statistica 10.0 software. The experiment was carried out in a completely randomized design. All data obtained from the experiment are expressed as mean ± standard error. Statistical differences were evaluated by using one-way ANOVA. P-values less than 0.05 were considered statistically significant.

## Results

*Pequi* oil had expressive amounts unsaturated fatty acids, especially oleic acid, a monounsaturated (MUFA), followed by the linoleic, a polyunsaturated acid (PUFA). Among saturated fatty acids (SFA), palmitic acid was the higher (Table 1). *Pequi* oil also presented 32.18 ± 8 mg/g of total carotenoids.

**Table 1:** Fatty acids profile of *pequi* oil (g.100 g^−1^)

Fatty acid	Carbon num.	Mean ± SD
Lauric	C12:0	0.04 ± 0.01
Myristic	C14:0	0.10 ± 0.01
Palmitic	C16:0	37.05 ± 0.04
Stearic	C18:0	2.12 ± 0.01
Arachidonic	C20:0	0.20 ± 0.01
Behenic	C22:0	0.07 ± 0.01
Lignoceric	C24:0	0.09 ± 0.01
Palmitoleic	C16:1	0.82 ± 0.01
Oleic	C18:1	57.42 ± 0.03
Linoleic	C18:2	1.38 ± 0.01
α-Linolenic	C18:3	0.32 ± 0.01
Eicosenoic	C20:1	0.25 ± 0.01
Total saturated	---	39.73 ± 0.03
Total unsaturated	---	60.27 ± 0.03

In the rat study, at first, *pequi* oil intake did not affect body weight and food intake, as well as FER and EER (Table 2).

**Table 2:** General characteristics of experimental groups after 15 weeks of treatment

Variables	C	PO
Body weight (g)	286.4 ± 55.9	277.9 ± 35.0
Food intake (g)	2209.5 ± 237.0	2406.3 ± 230.7
Energy intake (Kcal)	7247.3 ± 895.5	8373.8 ± 802.9
Feed efficiency ratio (g.g^−1^)	0.10 ± 0.02	0.09 ± 0.01
Energy efficiency ratio (g. Kcal^−1^)	0.032 ± 0.006	0.027 ± 0.004

*C* chow, *PO* Chow added *pequi* oil; Values are expressed as mean ± standard error. * indicates statistical difference (*p* < 0.05) between means by One way-ANOVA

We also found that hepatic triglycerides (TG) were reduced by *pequi* oil intake. All others cardiovascular risk factors were not changed (Table 3).

**Table 3:** Cardiovascular risk factors of experimental groups after 15 weeks of treatment

Variables	C	PO
Lee index (g/cm^3^)	3.1 ± 0.1	2.9 ± 0.2
Adiposity index (%)	2.9 ± 0.7	3.1 ± 0.7
Plama glucose (mg/dL)	123.7 ± 22.1	127.9 ± 9.6
Plasma insulin (ng/mL)	1.0 ± 0.2	1.1 ± 0.3
HOMA-IR	0.28 ± 0.05	0.27 ± 0.08
Plasma cholesterol (mg/dL)	58.1 ± 8.5	62.1 ± 6.6
Plasma triglycerides (mg/dL)	32.6 ± 5.3	33.8 ± 10.6
LDL-C (mg/dL)	14.0 ± 1.2	15.7 ± 1.5
HDL-C (mg/dL)	22.9 ± 5.9	23.0 ± 1.8
Hepatic cholesterol (mg.g^−1^)	5.9 ± 1.1	4.8 ± 1.6
Hepatic triglycerides (mg.g^−1^)	23.2 ± 2.4	17.0* ± 2.0
Systolic blood pressure (mmHg)	152.4 ± 21.8	150.7 ± 26.3
Heart Rate (bpm)	392.4 ± 62.2	402.7 ± 67.5
Double product index (mmHg x bpm)	60451.6 ± 16078.5	61586.5 ± 20209.6
Muscle mass index (mg.g^−1^)	4.6 ± 0.6	5.0 ± 0.7
Cardiomyocytes diameter (μm)	12.8 ± 0.8	13.1 ± 0.8

*C* chow, *PO* Chow added *pequi* oil; Values are expressed as mean ± standard error. * indicates statistical difference between means (*p* < 0.05) by One way-ANOVA

**Fig. 1:**
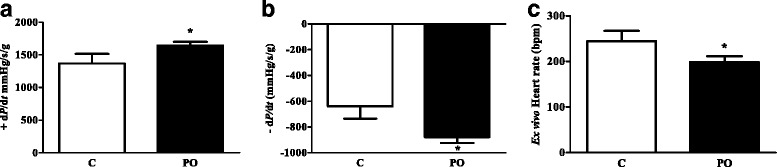
*Pequi* oil improves basal cardiac function and heart rate of rats. C = Chow and PO = Chow added *pequi* oil. Contractility index (+dP/dt) (**a**), relaxation index (−dP/dt) (**b**) and heart rate (**c**). Values are expressed as mean ± SD. * indicates statistical difference (*p* < 0.05) by One way-ANOVA

To address if the treatment with PO could directly alter cardiac function, we performed Langendorf analysis (Fig. 1). Rats showed increased basal cardiac function as evidenced by increased contractility (+dP/dt) [PO: 1640.7 ± 167.7 mmHg/s-1/g-1, C: 1366.9 ± 420.4 mmHg/s-1/g-1, Fig. 1a] and relaxation (−dP/dt) [PO: 878.5 ± 128.8 mmHg/s-1/g-1, C: 639.5 ± 271.3 mmHg/s-1/g-1, Fig. 1b] indexes. Furthermore, the intrinsic heart rate (PO: 198.3 ± 36.8 bpm, C: 244.3 ± 64.4 bpm) was lowered by *pequi* oil intake (Fig. 1c).

**Fig. 2:**
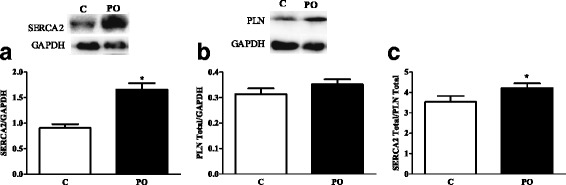
Pequi oil increases SERCA2a expression (**a**) and SERCA2a/PLB ratio (**c**) of rats. C = Chow and PO = Chow added pequi oil. Values are expressed as mean ± SD. * indicates statistical difference (*p* < 0.05) by One way-ANOVA

Since cardiac function was ameliorated by *pequi* oil, we investigated the involvement of key proteins with cardiac function modulation. We observed an increase in SERCA2a content in PO group (Fig. 2a). The same was not observed for PLB content (Fig. 2b). In addition, the SERCA2a/PLB ratio was also higher in PO group (Fig. 2c).

## Discussion

*Pequi* oil, accordingly to data available elsewhere [6, 38, 39], had oleic acid as its major fatty acid, being higher than olive oil, the main dietary source of it [40]. Otherwise, the second major fatty acid was palmitic, an important dietary SFA [41]. High carotenoid content was also observed. According to Rodriguez-Amaya et al. [42], to be a carotenoid source, a food must have more than 20 μg/g, which is associated to health benefits. We found 10× more carotenoids in *pequi* oil, so it could be an excellent carotenoid food source.

Thus, for the rat study, we decided to add *pequi* oil in the chow turning its lipid content 50% higher, which added oleic acid by 1.29 g.100^−1^ and carotenoids by 7.2 μg.g^−1^. Also, it increased palmitic acid by 0.83 g.100^−1^. According to Hariri and Thibault [9], it is necessary to increase lipid content of an experimental diet up to 30% of its total energy to induce metabolic disturbance in rodents. Adding *pequi* oil to the chow provided 18.51% of lipid energy.

Overall, our data indicated that long term *pequi* oil intake was able improve the ex vivo cardiac function, by increasing cardiac relaxation and contractility. We also inferred that this effect occurred independently of changes in systemic cardiovascular risk factor, since just hepatic TG was changed by *pequi* oil.

Indeed, the slightly higher lipid availability from *pequi* oil could have contributed to these results. To achieve a better cardiac function, it is necessary a correct oxygen supply and energy provision to meet the myocardium demands. Heart is known by its ability to produce energy from fatty acids because it is more capable to perform beta-oxidation, since it has high amounts and activity of enzymes related to. Heart ATP storage is limited and it can assure just a few seconds of beating. Because of that, cardiac muscle can adapt quickly to the energy demand and increases up to 100% its energy production from fatty acids, when there is higher availability of those nutrients [43].

Otherwise, the nature of the fatty acids in this oil may also have accounted to these changes. Palmitic acid, the second higher fatty acid in *pequi* oil, is oxidized rather than other fatty acids in heart [44]. Oleic acid, the major fatty acid in this oil, is able to up-regulate the transcription of genes coding for proteins involved in cardiac fatty acid transport and metabolism. These changes correspond to a 60% increase in cardiomyocyte fatty acid oxidation capacity [45].

Also, *pequi* oil intake provided exogenous antioxidants, especially carotenoids, which also could have accounted for those effects. Csepanyi et al. [19] showed in a rat model that carotenoid intake improved heart function at lower reperfusion times. These authors assigned those effects to the antioxidant properties of these compounds.

Conversely, although hepatic TG reduction was a timely result, a previous study from our lab also showed a significant reduction of those parameters in rats fed *pequi* pulp providing a 50% increase of dietary oil [38]. In addition, despite this result could not be related directly to cardiac function, there is a great body of evidence connecting hepatic lipid accumulation to cardiovascular risk, independently of coexisting cardiometabolic risk factors [46].

MUFA may exert their beneficial effects on hepatic fat content through their influence on lipid metabolism in the liver or in the abdominal adipose tissue [47]. A high MUFA diet could avoid hepatic lipid accumulation by activating catabolic pathways. It may result in degradation of the insulin-induced gene-1 protein, and therefore, inactivation of the transcription factor sterol regulatory element binding protein which promotes, among some effects, fat oxidation [40]. Indeed, an increasing body of evidence indicates an increment in fat oxidation rate, specifically with higher dietary MUFA levels, in several tissues [48, 49]. More recently, Liu et al. [13] showed in mice that hepatic oleic acid, provided both by diet or endogenously, is pivotal to prevent or to solve hepatic stress and inflammation induced by lipogenic diets.

Based on the systemic findings, we could infer that the improved cardiac function in *pequi* oil group was a consequence of intrinsic cardiac adaptations. The reduced heart rate and the increased cardiac SERCA2/PLB ratio in *pequi* oil group were important changes that can explain that. It is well established that a decrease in heart rate increases the diastolic period, which favors improved contractility/relaxation efficiency. Nevertheless, we believe that the improved cardiac function in *pequi* oil group was a consequence of the increased SERCA2a/PLB ratio.

It is known that, in rats, 92% of Ca^2+^ fluxes during cardiac excitation-contraction is regulated by SERCA2a. It acts as a sarcoplasmic reticulum (SR) protein regulated by PLB, facilitating SR calcium storage [50]. During systole, the action potential induces calcium release from SR and, the higher calcium availability, the higher contractility. During diastole, SERCA2a increases calcium reuptake to SR increasing the efficiency of relaxation [51]. In STZ-induced diabetic rats, the increase of SERCa2a expression protected from diabetic cardiomiopaty [52].

PLB is an integral SR membrane protein, which regulates SERCA2a activity. An upregulation in SERCA2a-to-PLB ratio is an important indicative of increased SERCA2a activity [53]. Thus, the increased SERCA2a/PLB ratio in *pequi* oil animal hearts may be an important mechanism that explain the increased cardiac contractility/relaxation index.

Still, our study shows some limitations. As there is no information about human intake of *pequi* oil as well as a few ones about doses used previously in animal models (as a whole food), it is possible that the amount of *pequi* oil added has not been sufficient to promote clearer effects, especially in systemic cardiometabolic risk factors. However, we chose to increase dietary lipids by 50% using *pequi* oil because we tried to associate some basic results gotten from pre-tests and in this way, the diet did not turned into a high fat [9]. Besides, our research group and others showed, previously, improvements in systemic cardiovascular risk factors in rats and humans fed *pequi* pulp or *pequi* oil pills providing 600 mg/d of *pequi* oil [23–25, 38, 39]. In this way. we would be offering at least, 600 mg/d of pequi oil. Also, we were unable to address the observed effects to MUFA, or carotenoids or both. However, at this point of investigation, we were more interested, in knowing if the whole food intake exerted any effect. In addition, a complete characterization of the mechanism and the reasons by which the long term intake of *pequi* oil led to lowering hepatic triglyceride deposition, bradycardia and increased SERCA2a/PLB ratio is beyond the scope of the present study and requires future investigations. Moreover, it is important to point out that this is the first paper showing cardiovascular effects of long-term *pequi* oil intake, especially on cardiac function.

Moreover, future research will be include more profound molecular analysis not only on calcium transient but also on the cardiac redox state [54] in hearts of animals feed *pequi* oil. It also would be necessary to evaluate if cardioprotective effects of *pequi* oil happens to be in other situations, such as, in different doses of *pequi* oil that mimic human servings and, or, in pathological conditions (non-alcoholic fatty liver disease, obesity, insulin resistance), or at minimal cardiometabolic disturbances, since food bioactive compounds may not show clear effects in healthy conditions.

## Conclusions

Taken together, our data indicates that *pequi* oil was able to improve rat ex vivo cardiac function, by increasing cardiac relaxation and contractility, despite no significant changes in systemic cardiovascular risk factors. The higher availability of lipids associated to the higher content of oleic and palmitic acids and carotenoids provided by *pequi* oil diet could be related, at least, in part to those findings.

## Abbreviations

C: Control diet
CHOL: Total cholesterol
GAPDH: Glyceraldehyde 3-phosphate dehydrogenase
GLU: Glucose
HDL-C: High-density lipoprotein
INS: Insulin
LDL-C: Low-density lipoprotein
MUFA: Monounsaturated fatty acids
PLB: Phopholamban
PO: *Pequi* oil diet
SERCA2a: Sarcoplasmic reticulum Ca2 + −ATPase
SFA: Saturated fatty acids
TG: Triglyceride

## References

[1] Go AS, Mozaffarian D, Roger VL, Benjamin EJ, Berry JD, Blaha MJ, et al. Executive summary: heart disease and stroke statistics—2014 Update: a report from the American Heart Association. Circulation. 2014;129(3):399–410. 10.1161/01.cir.0000442015.53336.12 24446411

[2] Lichtenstein AH, Appel LJ, Brands M, Carnethon M, Daniels S, Franch HA (2006). Diet and lifestyle recommendations revision 2006: a scientific statement from the American Heart Association of Nutrition Committee. Circ.

[3] Hooper L, Martin N, Abdelhamid A, Davey-Smith G (2015). Reduction in saturated fat intake for cardiovascular disease. Cochrane Database Syst Rev.

[4] Hammad S, Pu S, Jones PJ (2016). Current evidence supporting the link between dietary fatty acids and cardiovascular disease. Lipids.

[5] Schwab U, Lauritzen L, Tholstrup T, Haldorsson TI, Riserus U, Uusitupa M, et al. Effect of the amount and type of dietary fat on cardiometabolic risk factors and risk of developing type-2 diabetes, cardiovascular disease, and cancer: a systematic review. Food Nutr Res. 2014; 58. 10.3402/fnr.v58.25145. 10.3402/fnr.v58.25145 PMC4095759 25045347

[6] Lima A, Silva AMO, Trindade RA, Torres RP, Mancini-Filho J (2007). Composição química e compostos bioativos presentes na polpa e na amêndoa do pequi (*Caryocar brasiliense* Camb.). Rev Bras Frutic.

[7] Joris PJ, Mensink RP (2016). Role of cis-monounsaturated fatty acids in the prevention of coronary heart disease. Curr Atheroscler Rep.

[8] Bos M, de Vries J, Feskens E, van Dijk S, Hoelen D, Siebelink E (2010). Effect of a high monounsaturated fatty acids diet and a Mediterranean diet on serum lipids and insulin sensitivity in adults with mild abdominal obesity. Nutr Metab Cardiovasc Dis.

[9] Hariri N, Thibault L (2010). High-fat diet-induced obesity in animal models. Nutr Res Rev.

[10] Schwingshackl L, Hoffmann G (2012). Monounsaturated fatty acids and risk of cardiovascular disease: synopsis of the evidence available from systematic reviews and meta-analyses. Nutrients.

[11] Perdomo L, Beneit N, Otero YF, Escribano Ó, Díaz-Castroverde S, Gómez-Hernández A, Benito M (2015). Protective role of oleic acid against cardiovascular insulin resistance and in the early and late cellular atherosclerotic process. Cardiovasc Diabetol.

[12] Koeberle A, Shindou H, Harayama T, Shimizu T (2012). Palmitoleate is a mitogen, formed upon stimulation with growth factors, and converted to palmitoleoyl-phosphatidylinositol. J Biol Chem.

[13] Liu X, Burhans MS, Flowers MT, Ntambi JM (2016). Hepatic oleate regulates liver stress response partially through PGC-1α during high-carbohydrate feeding. J Hepatol.

[14] Airhart S, Cade WT, Jiang H, Coggan AR, Racette SB, Korenblat K (2015). A diet rich in medium-chain fatty acids improves systolic function and alters the lipidomic profile in patients with type 2 diabetes: a pilot study. J Clin Endocrinol Metab.

[15] Lopaschuk GD, Belke DD, Gamble J, Toshiyuki I, Schönekess BO (1994). Regulation of fatty acid oxidation in the mammalian heart in health and disease. Biochim Biophys Acta Lipids Lipid Metab.

[16] Azevedo-Meleiro CH, Rodriguez-Amaya DB (2004). Confirmation of the identity of the carotenoids of tropical fruits by HPLC-DAD and HPLC-MS. J Food Compost Anal.

[17] Cardoso LM, Reis BDL, Hamacek FR, Sant’Ana HMP (2013). Chemical characteristics and bioactive compounds of cooked pequi fruits (*Caryocar brasiliense* Camb.) from the Brazilian Savannah. Fruits.

[18] Maiani G, Periago-Castón MJ, Catasta G, Toti E, Cambrodón IG, Bysted A, Valoti M (2009). Carotenoids: actual knowledge on food sources, intakes, stability and bioavailability and their protective role in humans. Mol Nutr Food Res.

[19] Csepanyi E, Czompa A, Haines D, Lekli I, Bakondi E, Balla G (2015). Cardiovascular effects of low versus high-dose beta-carotene in a rat model. Pharmacol Res.

[20] Bezerra N, Barros T, Coelho N (2015). A ação do óleo de pequi (*Caryocar brasiliense*) no processo cicatricial de lesões cutâneas em ratos. Rev Bras Plantas Med.

[21] Palmeira SM, Silva PR, Ferrão JS, Ladd AA, Dagli ML, Grisolia CK (2016). Chemopreventive effects of pequi oil (*Caryocar brasiliense* Camb.) on preneoplastic lesions in a mouse model of hepatocarcinogenesis. Eur J Cancer Prev.

[22] Colombo N, Rangel M, Martins V, Hage M, Gelain D, Barbeiro D (2015). *Caryocar brasiliense* camb protects against genomic and oxidative damage in urethane-induced lung carcinogenesis. Braz J Med Biol Res.

[23] Miranda-Vilela AL, Lordelo GS, Akimoto AK, Alves PCZ, Silva-Pereira LC, Klautau-Guimaraes MN (2011). Genetic polymorphisms influence runners’ responses to the dietary ingestion of antioxidant supplementation based on pequi oil (*Caryocar brasiliense* Camb.): a before-after study. Genes Nutr.

[24] Miranda-Vilela A, Akimoto A, Alves P, Pereira L, Gonçalves C (2009). Dietary carotenoid-rich pequi oil reduces plasma lipid peroxidation and DNA damage in runners and evidence for an association with MnSOD genetic variant-Val9Ala. Genet Mol Res.

[25] Miranda-Vilela AL, Pereira LC, Gonçalves CA, Grisolia CK (2009). Pequi fruit (*Caryocar brasiliense* Camb.) pulp oil reduces exercise-induced inflammatory markers and blood pressure of male and female runners. Nut Res.

[26] Miranda-Vilela AL, Grisolia CK, Longo JPF, Peixoto RC, Almeida MC, Barbosa LCP (2014). Oil rich in carotenoids instead of vitamins C and E as a better option to reduce doxorubicin-induced damage to normal cells of Ehrlich tumor-bearing mice: hematological, toxicological and histopathological evaluations. J Nutr Biochem.

[27] Miranda-Vilela AL, Peixoto RC, Longo JPF, Portilho FA, Miranda KLC, Sartoratto PPC (2013). Dextran-functionalized magnetic fluid mediating magnetohyperthermia combined with preventive antioxidant pequi-oil supplementation: potential use against cancer. J Biomed Nanotechnol.

[28] AOCS (2009). A. O. C. S. Official methods and recommended practices of the AOCS.

[29] AOAC (2000). A.O.A.C. Official methods of analysis of AOAC International.17^th^ edittion.

[30] Novelli E, Diniz Y, Galhardi C, Ebaid G, Rodrigues H, Mani F (2007). Anthropometrical parameters and markers of obesity in rats. Lab Anim.

[31] Boustany CM, Bharadwaj K, Daugherty A, Brown DR, Randall DC, Cassis LA (2004). Activation of the systemic and adipose renin-angiotensin system in rats with diet-induced obesity and hypertension. Am J Physiol Regul Integr Comp Physiol.

[32] Matthews D, Hosker J, Rudenski A, Naylor B, Treacher D, Turner R (1985). Homeostasis model assessment: insulin resistance and β-cell function from fasting plasma glucose and insulin concentrations in man. Diabetologia.

[33] Folch J, Lees M, Sloane-Stanley G (1957). A simple method for the isolation and purification of total lipids from animal tissues. J Biol Chem.

[34] Schutte R, Thijs L, Asayama K, Boggia J, Li Y, Hansen TW, Ohkubo T (2013). Double product reflects the predictive power of systolic pressure in the general population: evidence from 9,937 participants. Am J Hypertens.

[35] Melo DS, Costa-Pereira LV, Santos CS, Mendes BF, Costa KB, Santos CFF (2016). Severe calorie restriction reduces cardiometabolic risk factors and protects rat hearts from ischemia/reperfusion injury. Front Physio.

[36] Almeida PWM, Gomes-Filho A, Ferreira AJ, Rodrigues CEM, Dias-Peixoto MF, Russo RC (2009). Swim training suppresses tumor growth in mice. J Appl Phys.

[37] Bradford MM (1976). A rapid and sensitive method for the quantitation of microgram quantities of protein utilizing the principle of protein-dye binding. Anal Biochem.

[38] Moreno LG, Oliveira LG, Melo DS, Costa-Pereira LV, Costa KB, Miranda JL, Rocha-Vieira E, Magalhães FC, Dias-Peixoto MF, Esteves EA (2016). *Caryocar brasiliense* fruit intake ameliorates hepatic fat deposition and improves intestinal structure of rats. J Med Plant Res.

[39] Teixeira TN, Esteves EA, Oliveira LG, Oliveira MLP, Santana RC, Rodrigues AP (2013). *Caryocar brasiliense* pulp increases serum HDL and reduces hepatic lipid accumulation in rats fed a high fat diet. J Med Plant Res.

[40] Lopez‐Miranda J, Delgado‐Lista J, Perez‐Martinez P, Jimenez‐Gómez Y, Fuentes F, Ruano J, Marin C (2007). Olive oil and the haemostatic system. Mol Nutr Food Res.

[41] Kien CL, Bunn JY, Stevens R, Bain J, Ikayeva O, Crain K (2014). Dietary intake of palmitate and oleate has broad impact on systemic and tissue lipid profiles in humans. Am J Clin Nut.

[42] Rodriguez-Amaya DB, Kimura M, Amaya-Farfan J (2008). Fontes brasileiras de carotenoides: Tabela brasileira de composição de carotenoides em alimentos.

[43] Kolwicz SC, Purohit S, Tian R (2013). Cardiac metabolism and its interactions with contraction, growth, and survival of cardiomyocytes. Circ Res.

[44] Longnus SL, Wambolt RB, Barr RL, Lopaschuk GD, Allard MF (2001). Regulation of myocardial fatty acid oxidation by substrate supply. Am J Physiol Heart Circ Physiol.

[45] van der Lee KA, Vork MM, De Vries JE, Willemsen PH, Glatz JF, Reneman RS (2000). Long-chain fatty acid-induced changes in gene expression in neonatal cardiac myocytes. J Lipid Res.

[46] Targher G, Bertolini L, Rodella S, Tessari R, Zenari L, Lippi G (2007). Nonalcoholic fatty liver disease is independently associated with an increased incidence of cardiovascular events in type 2 diabetic patients. Diabetes Care.

[47] Byrne CD, Targher G (2014). Ectopic fat, insulin resistance, and nonalcoholic fatty liver disease implications for cardiovascular disease. Arterioscler Thromb Vasc Biol.

[48] Bozzetto L, Prinster A, Annuzzi G, Costagliola L, Mangione A, Vitelli A (2012). Liver fat is reduced by an isoenergetic MUFA diet in a controlled randomized study in type 2 diabetic patients. Diabetes Care.

[49] Hussein O, Grosovski M, Lasri E, Svalb S, Ravid U, Assy N (2007). Monounsaturated fat decreases hepatic lipid content in non-alcoholic fatty liver disease in rats. World J Gastroenterol.

[50] Bers DM (2002). Cardiac excitation-contraction coupling. Nature.

[51] Frank KF, Bölck B, Erdmann E, Schwinger RHG (2003). Sarcoplasmic reticulum Ca2 + −ATPase modulates cardiac contraction and relaxation. Cardiovasc Res.

[52] Qi M, Xia H, Dai D, Dai Y (2006). A novel endothelin receptor antagonist CPU0213 improves diabetic cardiac insufficiency attributed to up-regulation of the expression of FKBP12.6, SERCA2a, and PLB in rats. J Cardiovasc Pharmacol.

[53] Kemi OJ, Wisloff U (2010). Mechanisms of exercise‐induced improvements in the contractile apparatus of the mammalian myocardium. Acta Physiol.

[54] He H, Shi M, Zenga X, Yanga J, Li Y, Wua L, Li L (2008). Cardioprotective effect of salvianolic acid B on large myocardial infarction mediated by reversing upregulation of leptin, endothelin pathways, and abnormal expression of SERCA2a, phospholamban in rats. J Ethnopharmacol.

